# Kavain suppresses human Aβ-induced paralysis in *C. elegans*

**DOI:** 10.17912/micropub.biology.000254

**Published:** 2020-05-21

**Authors:** Manish Chamoli, Shankar J Chinta, Julie K Andersen, Gordon J Lithgow

**Affiliations:** 1 Buck Instiute for Research on Aging, Novato, California, 94945 USA; 2 Touro University California, Vallejo, California, 94945 USA

**Figure 1:**
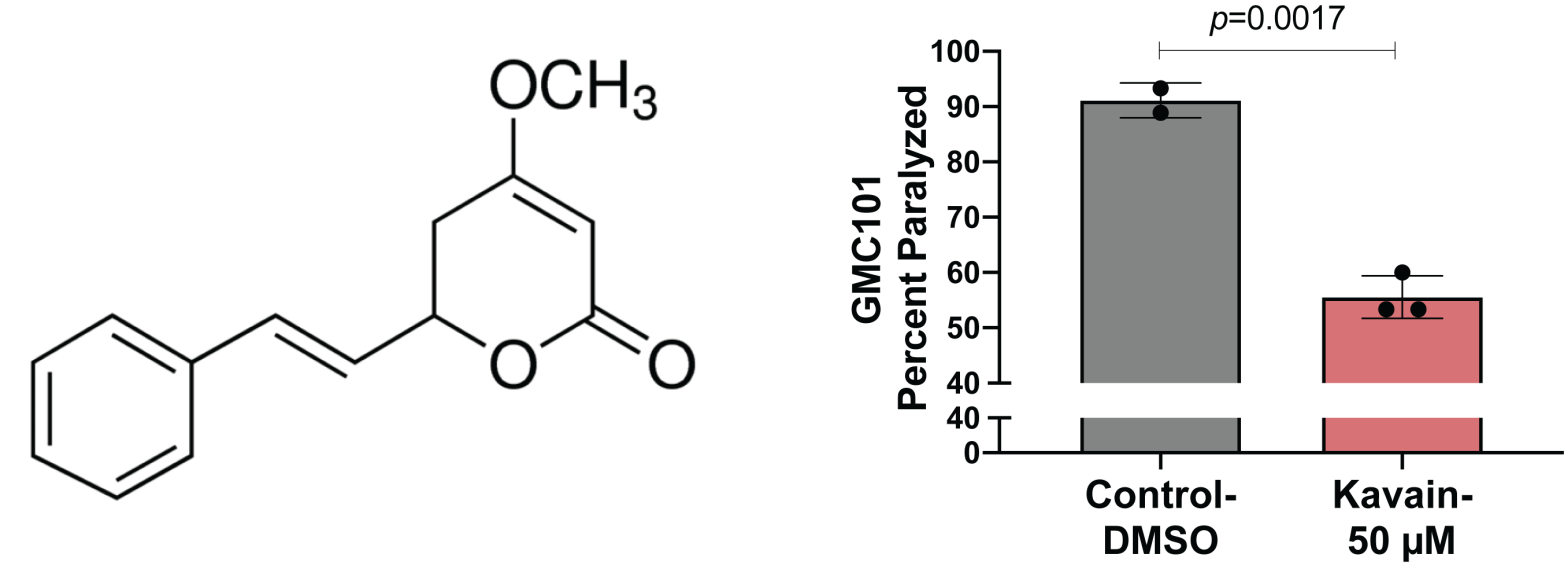
**Kavain suppresses human Aβ-induced paralysis in *C. elegans* (A)** Structure of kavain. **(B)** Aβ proteotoxicity in temperature-sensitive (enhanced paralysis at 25^o^C) GMC101 was determined by scoring the percent of animals paralyzed following kavain and Control-DMSO treatments. Data plotted as mean ± SD, n=45 animals per trial, Control-DMSO (N=90), Kavain-50 μM (N= 135), p-value calculated using unpaired Student’s t-test.

## Description

Kavain belongs to a group of lactone-based compounds collectively known as kavalactones, present in the pepper plant kava (*P. methysticum*). Kavalactones have been shown to possess diverse biological activities including sedation and anxiolysis (Ooi *et al.*, 2018). Kavain in particular has been demonstrated to show potent anti-inflammatory properties in various *in vitro* and animal models (Guo *et al.*, 2018; Singh *et al.*, 2018; Tang and Amar, 2016; Yuan *et al.*, 2011). A study in *C. elegans* reported that kavain increases lifespan by inhibiting advance glycation end-products (AGEs), which are known to suppress lifespan (Chaudhuri *et al.*, 2016; Upadhyay *et al.*, 2014). Another study reported that kavain increases acetylcholine (ACh) transmission at the neuromuscular junction (Kautu *et al.*, 2017). Since loss in ACh transmission and increased formation of AGEs are closely linked to Aβ-pathology, we hypothesized that kavain may protect against Aβ-induced toxicity (Kar *et al.*, 2004; Li *et al.*, 2013). We tested kavain in the *C. elegans* GMC101 strain that over-expresses human Aβ in body wall muscle cells (McColl *et al.*, 2012). Kavain at a concentration of 40 and 80 μM was shown to increase lifespan, thus we decided to use a dose between these ranges (Upadhyay *et al.*, 2014). We observed GMC101 animals fed 50 μM kavain showed significantly less paralysis when shifted to the higher permissive temperature (25^o ^C). The result shows that kavain suppresses Aβ-induced proteotoxicity*.*

## Methods

NGM agar plates (35 mm) were prepared under sterile conditions in a laminar flow hood at room temperature (22^o^C). To these plates, 100 μl of *E.coli* OP50 was added to form a circular bacterial lawn on the center of each plate. Plates were then left inside the hood (lid closed) for drying. A 100 mM stock of compound was prepared in 100% DMSO and stored in small aliquots at -20°C. From the stock solution, 130 µl of the working drug (50 μM) or control-DMSO (0.05%) solution was prepared by mixing 1.5 μl of stock solution with 130 µl of sterile water and adding to the top of the 35 mm NGM plates (with 3 mL NGM agar) 48 hours post-bacterial seeding. Compound was distributed over the entire plate surface and allowed to dry in a sterile hood with the lid open for at least 1 hour. Plates were then allowed to sit at 20°C for 24 hours before use.

Egg-lay synchronized populations of GMC101 animals were grown from eggs at 20^o ^C until the L4 larval stage and then transferred to fresh 35 mm plates treated with Control-DMSO plates or kavain-50 μM. Plates were immediately shifted to 25^o^C and paralysis was scored 48 hours after the temperature shift. Animals were scored as paralyzed if they failed to move during observation and exhibited ‘halos’ of cleared bacteria around their heads (indicative of insufficient body movement to access food), eggs accumulated close to the body, or if they failed to respond to touch-provoked movement with a platinum wire.

## Reagents

GMC101: *dvIs100* [unc-54p::Aβ-1-42::unc-54 3′-UTR + mtl-2p::GFP]. [unc-54p::Aβ-1-42] expresses full-length human Aβ_1-42_ peptide in body wall muscle cells that aggregates *in vivo*. Kavain: Sigma Aldrich (#59780).
